# Reply to: “Hysterectomy versus continuing conservative management: which is better for disseminated intravascular coagulation?”; Shinya Matsuzaki, MD, PhD, Yoshikazu Nagase, MD, Masayuki Endo, MD, PhD, Tadashi Kimura, MD, PhD

**DOI:** 10.1007/s00404-021-05964-5

**Published:** 2021-02-05

**Authors:** C. Biele, L. Kaufner, A. Nonnenmacher, K. von Weizsäcker, M. Z. Muallem, W. Henrich, Thorsten Braun

**Affiliations:** 1 Department of obstetrics (or anesthesiology or genecology…), Charité – Universitätsmedizin Berlin, corporate member of Freie Universität Berlin, Humboldt-Universität zu Berlin, and Berlin Institute of Health, Augustenburger Platz 1, 13353 Berlin, Germany; 2 Department of Anesthesiology and Operative Intensive Care Medicine, Charité-Universitätsmedizin Berlin, Freie Universität Berlin, Humboldt-Universität zu Berlin, and Berlin Institute of Health, Berlin, Germany; 3 Department of Gynecology with Center for Oncological Surgery, Charité – Universitätsmedizin Berlin, corporate member of Freie Universität Berlin, Humboldt-Universität zu Berlin, and Berlin Institute of Health, Berlin, Germany; 4 Department of ‘Experimental Obstetrics’ and Study Group ‘Perinatal Programming’, Charité – Universitätsmedizin Berlin, corporate member of Freie Universität Berlin, Humboldt-Universität zu Berlin, and Berlin Institute of Health, Berlin, Germany

We would like to thank Matsuzaki S. et al. for reading and valuables comments to our manuscript „Conservative management of abnormally invasive placenta complicated by local hyperfibrinolysis and beginning disseminated intravascular coagulation“ [1].

We support the assumption that severe cases are possible more often treated conservatively (expectant management, EM) and are probably also more often reported in the literature. This is not owed to selection or publication bias, but to the fact that EM is a recommended treatment option especially for severe cases of placenta percreta with organ invasion, when surgical management is considered very high risk as reviewed and recommended recently in the IS-PAS management guidelines [3, 13]. Regarding the incidence of DIC (disseminated intravascular coagulation) in the different groups of placenta accreta spectrum (PAS) during EM, no statement so far can be made, considering that there are only 12 reported cases in the literature [1].

Second, we entirely consent on this point, as our concluding remarks are presented. Therapy for DIC remains unclear, since there are only three reported cases so far [1, 8, 11]. Oral dosage of tranexamic acid might be a promising therapy concept and should be subject of future studies [1, 11].

Third, the authors ask one of the fundamental questions regarding therapy of PAS, whether (cesarean) HE or EM is the better option of treatment, in general and in the case of DIC.

The authors suggest three considerations for deciding whether to continue EM or to perform HE in case of DIC:

Asymptomatic or symptomatic DIC. According to the authors continuation of EM might only be an option in cases of asymptomatic DIC.

Up to now there are three cases of successful treatment of DIC, where HE was not required. In one case the patient was asymptomatic [8], in two cases the patients presented with symptoms (vaginal bleeding, gingival bleeding, easy bruising) [1, 11]. Based on these results we cannot agree with this statement.

Expected success rate of conservative management is > 90% after nine weeks of cesarean delivery [7]. The authors conclude this in their review, whereas this applies to all patients, not only those with DIC. Seven patients in the review developed DIC, the median time to occurrence was 67 days after cesarean section (CS), which is 9.5 weeks. Six of the seven patients underwent HE, without information on which day postpartum it was performed. Based on this information we cannot agree with this point either.

The severity of PAS. Regarding the letter to the editor, the success rate for EM was higher in non-percreta cases than in percreta cases. On the other hand, the authors recommend HE in non-percreta cases, although it is not clear whether this is a general recommendation or refers to cases with DIC during EM. Further they refer to the high surgical morbidity of HE during EM of placenta percreta. Taking this information into account, it remains unclear whether a severe case of PAS hints for or against the continuation of EM in cases with DIC according to the authors.

Regarding EM of PAS in general the authors report a success rate of 61.8% for uterine preservation [7], other available reviews report success rates from 58 to 93% [2, 9, 10, 12, 15]. A retrospective cohort analysis comparing the outcomes of patients having received cesarean HE (*n* = 20), conservative surgery (placental removal without HE, *n* = 11) and EM (*n* = 15) shows a success rate of 93% regarding uterine preservation in the two conservative groups and no significant difference between the two groups. Patients from the EM group experienced significantly lower blood loss and less need for blood transfusion than patients of the other groups [6]. The reported morbidity rate in EM of PAS was 6% [12].

Taking all available data into account, EM might be a safer alternative to cesarean HE in the treatment of PAS. On the other hand, unpredictable and severe complications (infection, hemorrhage, DIC) are possible [14]. The current expert consensus is, that the question of the best therapy option cannot be clarified on the basis of current data and patients should be counselled on both options including the risks. EM seems to be an alternative to cesarean HE especially for patients with unfinished family planning or cases of severe placenta percreta with organ invasion [3, 13]. However, no unified classification of PAS is reported so far in those studies and a direct comparison between studies is difficult. FIGO published in 2019 a new PAS grading classification [4] and recently Eric Jauniaux recommended a histopathological classification of PAS to use [5]. A unified grading system will hopefully help in the future to better compare management strategies and outcomes from centers worldwide and further, high-quality studies are essential to finally clarify this question.

## Data Availability

Not applicable.
